# Supplementary material for: Galectin-1 Modulates the Fusogenic Activity of Placental Endogenous Retroviral Envelopes
Source: Viruses. 2023 Dec 16;15(12):2441. doi: 10.3390/v15122441 (PMC10747188; doi:10.3390/v15122441)
Supplement: Supplementary file 1 [file viruses-15-02441-s001.zip › Supplementary Figure 1.pdf]

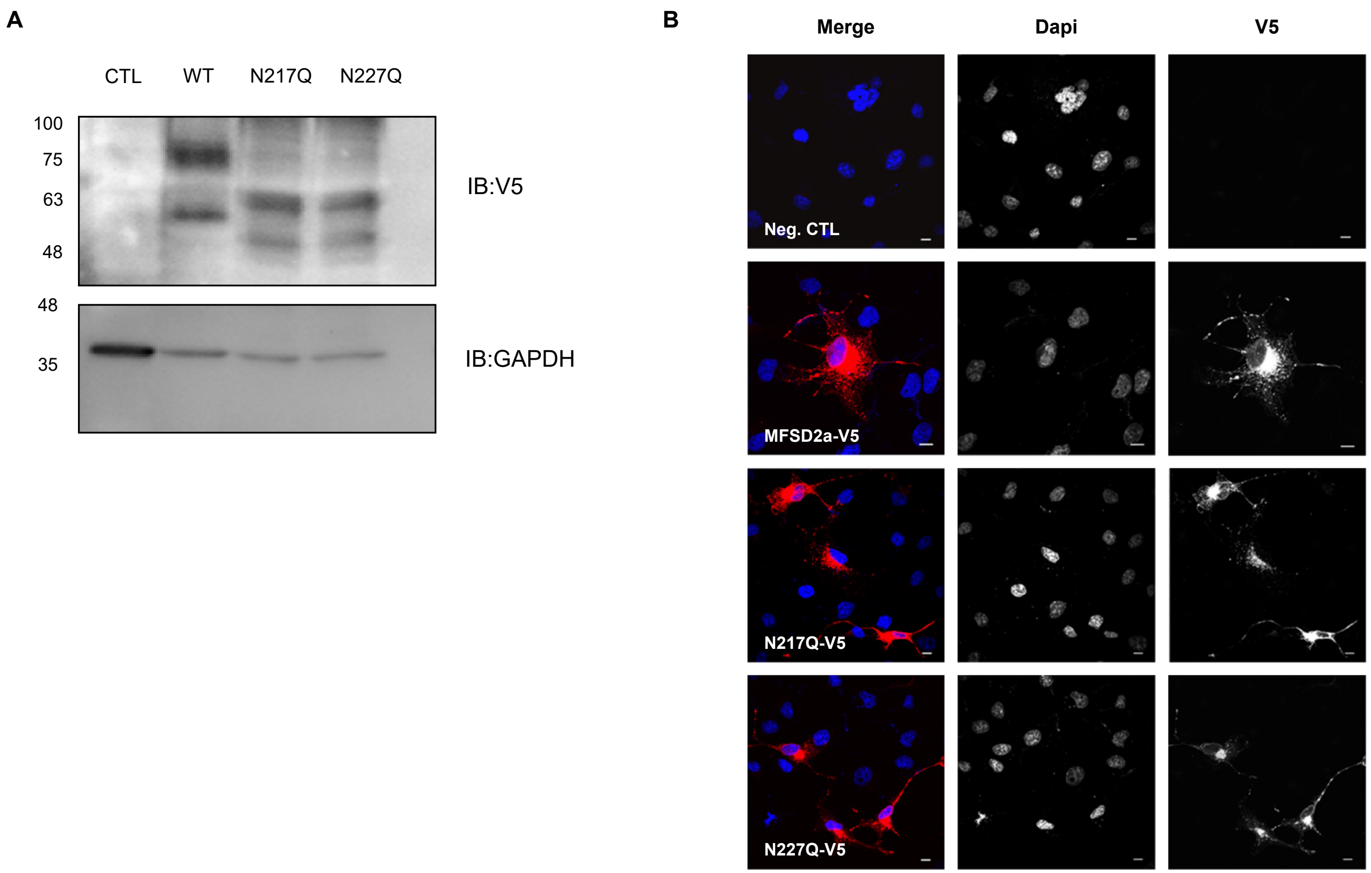


**Supplementary Figure 1**: Expression and localisation of MFSD2a N-glycosylation mutants in HEK293T cells. **A**. HEK293T cells were transfected with pLVX-YFP (CTL), pLVX-MFSD2a-V5 (WT), pLVX-N217Q-V5 or pLVX-N227Q-V5. Cells were lysed 48 h after transfection and protein extracts were analysed by Western blot using an anti-V5 antibody (upper panel). GAPDH detection served as a loading control (lower panel). **B**. Cos-7 cells were transfected with pLVX- (neg. CTL), pLVX-MFSD2aV5, pLVX-N217QV5 or pLVX-N227QV5. Cells were fixed 48 h after transfection and MFSD2a was detected using anti-V5 and Alexa Fluor 568-conjugated anti-mouse IgG antibodies (red) while nuclei were stained with DAPI (blue). Stained cells were analysed by confocal microscopy using a Nikon Eclipse Ti microscope coupled with a Nikon A1R confocal unit and the CFI Plan Apochromat λ 60x/1.4 oil objective. Representative images of three independent transfections are shown. Scale bar: 10 μm.
